# PRC2 Regulated Atoh8 Is a Regulator of Intestinal Microfold Cell (M Cell) Differentiation

**DOI:** 10.3390/ijms22179355

**Published:** 2021-08-28

**Authors:** Joel Johnson George, Laura Martin-Diaz, Markus J. T. Ojanen, Rosa Gasa, Marko Pesu, Keijo Viiri

**Affiliations:** 1 Faculty of Medicine and Health Technology, Tampere University Hospital, Tampere University, 33520 Tampere, Finland; joel.george@tuni.fi (J.J.G.); Laura.martindiaz@tuni.fi (L.M.-D.); markus.ojanen@tuni.fi (M.J.T.O.); marko.pesu@tuni.fi (M.P.); 2 Diabetes and Obesity Research Laboratory, Institut D’investigacions Biomèdiques August Pi I Sunyer (IDIBAPS), Center Esther Koplowitz C/Rosselló, 149-153 Barcelona, Spain; rgasa@clinic.cat

**Keywords:** gut immunity, M cells, Peyer’s patch, RankL, transcytosis

## Abstract

Intestinal microfold cells (M cells) are a dynamic lineage of epithelial cells that initiate mucosal immunity in the intestine. They are responsible for the uptake and transcytosis of microorganisms, pathogens, and other antigens in the gastrointestinal tract. A mature M cell expresses a receptor Gp2 which binds to pathogens and aids in the uptake. Due to the rarity of these cells in the intestine, their development and differentiation remain yet to be fully understood. We recently demonstrated that polycomb repressive complex 2 (PRC2) is an epigenetic regulator of M cell development, and 12 novel transcription factors including Atoh8 were revealed to be regulated by the PRC2. Here, we show that Atoh8 acts as a regulator of M cell differentiation; the absence of Atoh8 led to a significant increase in the number of Gp2+ mature M cells and other M cell-associated markers such as Spi-B and Sox8. In vitro organoid analysis of RankL treated organoid showed an increase of mature marker GP2 expression and other M cell-associated markers. Atoh8 null mice showed an increase in transcytosis capacity of luminal antigens. An increase in M cell population has been previously reported to be detrimental to mucosal immunity because some pathogens like orally acquired prions have been able to exploit the transcytosis capacity of M cells to infect the host; mice with an increased population of M cells are also susceptible to *Salmonella* infections. Our study here demonstrates that PRC2 regulated Atoh8 is one of the factors that regulate the population density of intestinal M cell in the Peyer’s patch.

## 1. Introduction

The gastrointestinal tract is subject to constant exposure to antigens, microorganisms, and foreign pathogens. The mucosal lining of the intestinal tract employs multiple mechanisms for immunosurveillance, which include epithelial tight junctions, production of antimicrobial peptides by Paneth cells, mucins from goblet cells, innate antigen receptors, and acquired immunity in the form of secretory IgA [1]. Phagocytic and transcytosis capabilities are critical to inducing an antigen-specific immune response. For this, the mucosal immune system is organized into inductive tissues such as the gut-associated lymphoid tissue (GALT), these include a specialized region known as Peyer’s patches (PPs) in the intestine. PPs are covered by dome-shaped follicle-associated epithelium (FAE) which are composed of specialized intestinal epithelial cells (IECs) known as Microfold cells (M cells) [2,3].

M cells are phagocytic epithelial cells that enable the uptake and transcytosis of luminal antigens into the GALT, they are responsible for the rapid transport of bacterial antigens to antigen-presenting immature dendritic cells [4,5]. M cells display characteristically different morphology from neighboring epithelial cells. On their apical side, they have irregular, short microvilli, while on their basolateral side they have an M-shaped pocket structure that houses antigen-presenting cells such as macrophages, B cells, and dendritic cells [6,7,8,9]. Mature M cells express the receptor Glycoprotein 2 (Gp2), critical for the uptake of *Salmonella typhimurium* and several other pathogenic antigens [10]. Consequently, mice lacking M cells or Gp2 receptors exhibit profound deficiencies in immune response as demonstrated by a decline in the production of antigen-specific secretory IgA (SIgA) in the gut and impaired antigen-specific T cell responses in mice infected with *Salmonella typhimurium* [5,10,11]. 

M cells arise from cycling intestinal stem cells in the crypts (which express Lgr5), but they are predominantly localized in the FAE due to the stimulation by nuclear factor κ B ligand (RankL) [12,13]. RankL is secreted from specialized stromal cells under the FAE which are known as M cell inducer cells [14]. RankL binds to Rank receptors on the Lgr5+ cells to activate TRAF6, which in turn leads to a signaling cascade of NFκB signaling, both classical and non-canonical [15]. Spi-B and Sox8 were identified as important transcription factors necessary for differentiation and functionality of M cells; Spi-B null and Sox8 null mice showed a lack of M cells and severely impaired transcytosis capabilities [16,17]. However, neither Spi-B nor Sox8 was sufficient for Gp2 expression as seen in both Spi-B null mice with intact Sox8 activation and Sox8 null mice with intact Spi-B activation. Although the progenitor cells approaching the FAE are constantly stimulated by activating signals, only ~10–20% of the cells in FAE are M cells. This suggests the presence of a regulatory mechanism in the FAE that prevents all the cells from differentiating into M cells. Osteoprotegerin (OPG) is a soluble decoy receptor for RankL, and it plays a regulatory role in maintaining the M cell density in the intestine by competing with Rank for binding to RankL. OPG null mice exhibited an increase in functionally mature M cell [18]. Despite the important role that M cells play in initiating mucosal responses, the mechanism for M cell development has yet to be fully characterized.

To further understand the differentiation and development of M cells, we had previously performed Chip-seq and Gro-seq for M cells and discovered 12 transcription factors that were epigenetically regulated (six upregulated and six silenced) [19]. One of the genes upregulated in our analysis was *Atoh8*. Here, we find that Atoh8 plays a critical role in regulating the differentiation of M cells; Atoh8 was expressed exclusively in M cells in the Peyer’s patches and was critical to maintaining the density of M cells in the FAE. Moreover, intestinal-specific Atoh8 deletion showed an increased capacity for transcytosis. Overall, our findings show that Atoh8 plays a homeostatic role in maintaining the M cell population and that it limits the translocation of invasive antigens from the luminal side into Peyer’s patches. 

## 2. Results

### 2.1. Atoh8 Is Expressed in M Cells and Induced by RankL-Rank Signaling

**Figure 1:**
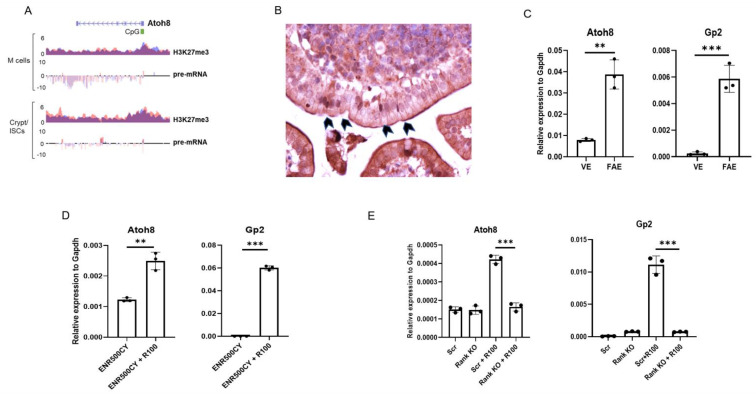
Atoh8 is expressed in FAE in Peyer’s patches and is dependent on Rank-RankL signaling. (**A**) H3K27me3 occupancy at CpG islands spanning the promoter and first exon of the *Atoh8* gene in organoids treated with RankL (M cells) or treated with Wnt3a and Chir99021 (Crypt/ISCs). Below, pre-mRNA expression of *Atoh8* in organoids treated as above (y-axis: normalized tag count, ENR500 = R-spondin 500 ng/mL, R100 = Rankl 100 ng/mL). (**B**) Section of PP from wild type mice stained with Atoh8 antibody. Arrowheads indicating Atoh8 expression in the nuclei of M cells in FAE. (**C**) RT-qPCR analysis of *Atoh8* and *Gp2* in the FAE and VE from C57BL/6JRj mice (*n* = 3). (**D**) Organoids generated from wild type mice were stimulated with 100 ng of RankL for 4 d. *Atoh8* and *Gp2* expression was examined by RT-qPCR analysis. (**E**) Rank KO organoids and Scrambled organoids generated by lentiCRISPR v2 were incubated with RankL for 4 days, *Atoh8* and *GP2* expression was analyzed by RT-qPCR. In panels (**C**–**E**), unpaired two-tailed Student’s *t*-test was performed for three independent experiments, *** *p* < 0.005; ** *p* < 0.01.

In our previous study, we sought to identify how PRC2 regulates M cell differentiation and discovered 12 transcription factors that were PRC2-regulated specifically in M cell differentiation (six upregulated and six silenced) [19]. *Atoh8* turned up as one of the RankL-induced PRC2 regulated genes (log2 fold changes −3.15 RankL vs WENRC and -2.58 RankL vs ENRI) (Figure 1A). Here, immunohistochemistry analysis of murine PPs revealed that Atoh8 is localized in the nuclei of cells in the Peyer’s patches (Figure 1B). RNA was isolated from the FAE and villous epithelium, and RT-qPCR analysis confirmed that Atoh8 was significantly enriched in the Peyer’s patches (Gp2 as a marker) when compared to the villi (Figure 1C). Mouse intestinal organoids isolated from the crypts were treated with RankL for 4 days, *Atoh8* was observed to be significantly upregulated along with GP2, confirming that Atoh8 is induced by RankL signaling (Figure 1D). Rank-deficient mouse intestinal organoids were generated using the Lenti V2 CRISPR/Cas9 system; RankL treatment did not induce *Atoh8* expression, suggesting that *Atoh8* expression falls under the purview of Rank-RankL signaling (Figure 1E). Validation of RANK KO was confirmed via immunoblot analysis (Supplementary Figure S1A). 

Previously in osteoclast, it was shown that BMP-induced Atoh8 regulated the RankL/OPG ratio indirectly via Runx2 to regulate osteoclast number and maintain bone volume in mice [20]. Our Gro-seq analysis of RankL organoids from our previous paper revealed that RankL signaling did lead to an upregulation of *BMP2* and *BMP6* but not *Runx2* [19]. The *BMP6* and *BMP2* activation by RankL was confirmed by qPCR analysis (Supplementary Figure S3A). Furthermore, treating intestinal organoids in EGF, R-spondin and 100 ng/mL of BMP2 and BMP6 showed that *Atoh8* could be induced by BMP2 and BMP6 alone (Supplementary Figure S3C,D).

### 2.2. Atoh8 Deficiency Augments M Cell Differentiation along with Other M Cell-Associated Transcription Factors

**Figure 2:**
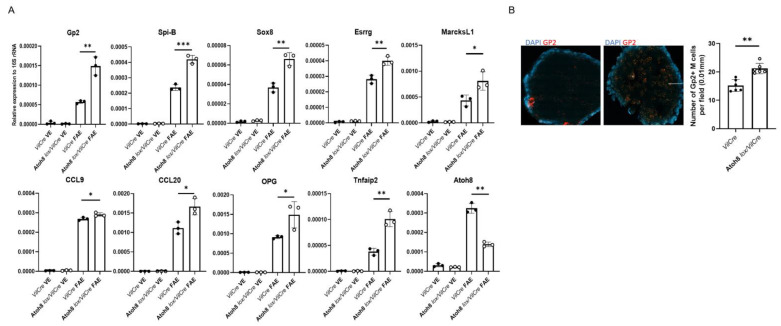
The loss of Atoh8 increases the number of mature M cells in the FAE. (**A**) qPCR analysis of M cell-associated genes in the FAE and VE of Atoh8 *lox/VilCre* and *VilCre* mice. ***, *p* < 0.005, *, *p* < 0.05 **, *p* < 0.01; n.s., not significant; unpaired two-tailed Student’s *t*-test, *n* = 3. (**B**) Whole-mount immunostaining of PPs from *VilCre* and Atoh8 *lox/VilCre* mice. The number of GP2+ M cells per field of FAE (0.01 mm2) was compared between them (*n* = 8). Scale bars, 200 μm.

Since our previous and current data demonstrate that *Atoh8* is a PRC2-regulated gene in M cell differentiation, located in PPs and induced by RankL, we hypothesized that Atoh8 may contribute to M cell differentiation. Intestine-specific Atoh8 knockouts were generated by crossing Atoh8 *lox/lox* with *VilCre* mice (from now on referred to as Atoh8 *lox/VilCre*). M cells were analyzed from the GALT of Atoh8 *lox/VilCre* mice. RT-qPCR analysis of RNA isolated from the FAE of Atoh8 *lox/VilCre* mice showed an increase in the expression of mature GP2+ M cells when compared to the control *VilCre* mice (Figure 2A). Spi-B and Sox8, which are transcriptional factors critical for functional M cell development, were markedly higher in the Atoh8 *lox/VilCre* mice (Figure 2A). Esrrg which is previously known to be essential for the development of M cells was also shown to be upregulated (Figure 2A). Early expression markers of M cells such as *CCL20*, *MarcksL1*, and *TNFAIP2* were also upregulated (Figure 2A). *OPG*, the decoy receptor for RankL was also showed higher expression in the Atoh8 *lox/VilCre* mice. The increase in Gp2+ M cells was also corroborated with whole-mount immunostaining (Figure 2B). We observed increased Gp2+ cells in the Atoh8 *lox/VilCre* mice, counting 22.4 ± 2.85 cells/0.01 mm in comparison with control *Vil/Cre* mice that had 15± 0.09 cells/0.01 mm.

### 2.3. Atoh8 Deficiency Does Not Affect the B and T Cell Composition of Peyer’s Patches

**Figure 3:**
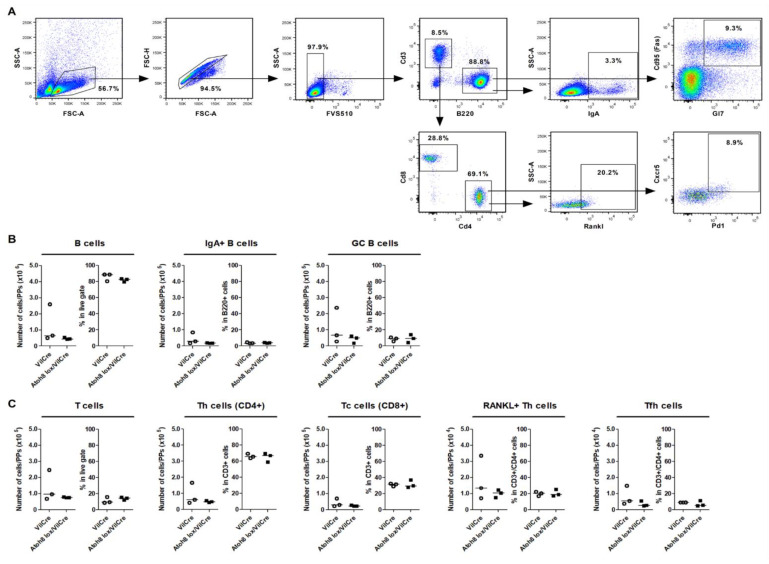
The loss of Atoh8 did not change the composition of lymphoid cells in 4-week-old mice after weaning. (**A**) Gating scheme for the analysis of B and T cell populations in *VilCre* and Atoh8 *lox/VilCre* ileal PPs. B (CD3ε−B220 + ) cells were analyzed for IgA+ B and CD95+GL7+ GC B cells. T (CD3ε+B220−) cells were analyzed for total CD4+ Th, CD8α+ cytotoxic T (Tc), and CD4+CD8α−CXCR5+PD-1+ Tfh cells. (**B**,**C**) FlowJo analysis of indicated immune cells in ileal PPs. (**B**) Number of total B cells, GC B cells, and IgA+ B cells in 100,000 recorded cells. (**C**) Number of total T cells, Tc cells, Th cells, and Tfh cells in 100,000 recorded cells; Student’s *t*-test, *n* = 3 per group.

Germinal centers (GC) in the GALT are important structures in which mature B cells proliferate and undergo somatic hypermutation and class switch recombination mediated by the follicular T helper cells (Tfh cells) [21,22]. Knockdown of transcription factors necessary for M cell differentiation like Spi-b and Sox8 leads to a lack of Gp2+ cells as well as causes a reduction in B and T cell populations in the Peyer’s patch [16,17]. OPG is another gene upregulated in M cell development. The gene transcribes a soluble decoy receptor that binds to RankL. Knockdown of OPG exhibited an increase in the M cell population as well as an increase in lymphoid cells that are involved in mucosal response [18]. Consequently, the increase in functionally mature M cells in Atoh8 *lox/VilCre* mice could affect both B and T cell populations compared to control animals even in steady state. However, similar populations of total B and T cells were observed in the PPs in Atoh8 *lox/VilCre* mice compared to controls, and we did not see any increase in the amount of IgA+ B cells at 4 weeks of age (Figure 3A–C). Furthermore, we also looked at follicular T helper cells (Tfh cells) as they promote germinal center reactions and affinity maturations to produce high affinity IgA [23]. Tfh cells and GC B cells had similar populations in *VilCre* and Atoh8 *lox/VilCre* (Figure 3C), and the amount of RankL producing Th cells was unaltered in both sets of mice. Overall, the lack of Atoh8 does not affect the B and T cell subpopulations or the quantities of IgA-producing B cells and Rankl+ T cells in pathogen-free mice.

### 2.4. Epithelium Intrinsic Atoh8 Is Responsible for the Increase in M Cell Population

**Figure 4:**
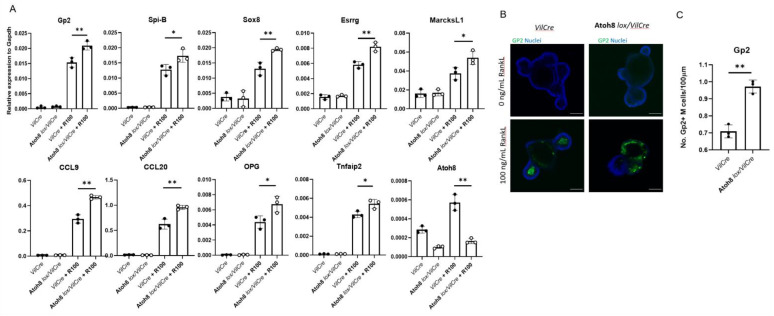
Increase in M cell maturation in Atoh8 *lox/VilCre* results from epithelium-intrinsic defect. (**A**,**B**) Organoids from small intestinal crypts of Atoh8 *lox/VilCre* and *VilCre* mice were cultured with or without RANKL for 3 d. (A) qPCR analysis of M cell-associated genes expressed in the organoid cultures. Values are presented as the mean ± SD; **, *p* < 0.01; *, *p* < 0.05; n.s., not significant; unpaired two-tailed Student’s *t*-test, *n* = 3. Data are representative of two independent experiments. (**B**) Immunostaining images for GP2 (green) on organoids. Bars, 100 µm. (**C**) The number of Gp2+ M cells and Sox8+ M cells per length epithelium of organoids were compared between *VilCre* and Atoh8 *lox/VilCre* treated organoids (*n* = 3). Images are representative of three independent experiments.

The subepithelial dome is an afront to multiple signaling pathways, therefore it was necessary to validate if Atoh8 regulation of M cell development was epithelium intrinsic. Mouse intestinal organoid culture isolated from crypts of Atoh8 *lox/VilCre* and *control VilCre* mice were treated for 4 days with 100 ng/mL of RankL to induce M cell differentiation. *Spi-B*, *Sox8*, and *Esrrg* were observed to be significantly upregulated when compared to the control. The epithelial cells mimicked the significantly increased Gp2 expression as observed in vivo in the FAE as well as the early markers expressions of *CCL9*, *Marcksl1*, and *TNFAIP2* (Figure 4A). Immunostaining images for Gp2 on intestinal organoids treated with RankL showed higher expression for Atoh8 *lox/VilCre* organoids compared to *VilCre* organoids (Figure 4B). Taken together, these data indicate that epithelial intrinsic Atoh8 is sufficient to regulate M cell differentiation.

### 2.5. Atoh8 Deficiency Leads to Increased Transcytosis Capacity

**Figure 5:**
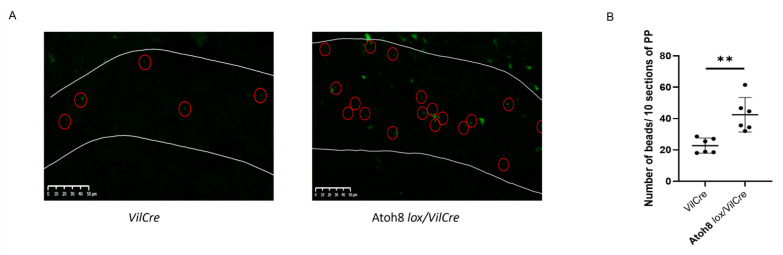
The loss of Atoh8 causes increased uptake of luminal nanoparticles into the follicle. (**A**,**B**) Green fluorescent latex beads (200 nm diameter) were orally administered to *VilCre* or Atoh8 *lox/VilCre* mice. 3 h later, PPs were collected from the jejunum. Ten consecutive cryosections of each PPs were examined by fluorescence microscopy, and the number of particles was counted manually. (**A**) Representative images of cryosections. White lines indicate FAE. Red circles indicate fluorescent particles. Bars, 50µm. (**B**) Quantification of particles in PPs. **, *p* < 0.01; Student’s *t*-test; *n* = 3 per genotype.

Gp2+ M cells in the Peyer’s patches are essential for transcytosis of antigens and foreign particles to initiate a mucosal immune response. As Atoh8 knockout mice presented more Gp2 cells in the FAE, we investigated if these Gp2 cells are functional. To explore this, we administered orally fluorescent nanoparticles to Atoh8 *lox/VilCre* and control *VilCre* mice. Four hours after the oral administration, PP’s from both mice were removed and the number of particles transcytosed in each PP was counted with a fluorescence microscope (Figure 5A). We observed that the uptake of nanoparticles in the Atoh8 *lox/VilCre* Peyer’s patch was increased significantly by over 2-fold when compared to the *control* mice (Figure 5B).

## 3. Discussion

M cells are Intestinal epithelial cells specialized in gut immunity and transcytosis of antigens via GP2 to initiate immune responses. Previous research has revealed that M cell differentiation requires activation of Nf-κB signaling, activation of transcription factors Spi-B, Esrrg, and Sox8, membrane-bound RankL from lymphoid cells, and S100A4 from Dock8 cells [15,16,17,24]. However, other factors controlling M cell developments remain to be elucidated. For our analysis into M cell differentiation, we had previously performed a Chip-seq and Gro-seq of M cells and our data set revealed that PRC2 regulated 12 novel transcription factors (six silenced and six upregulated) in M cell development [19]. Atoh8 was one of the six transcription factors upregulated by the PRC2. Here, we describe that M cells in the FAE express Atoh8, specifically induced by RankL treatment in intestinal organoids differentiating to M cells and under the purview of the RankL-Rank signaling pathway.

Atoh8 belongs to group A of the bHLH transcription factor family. Members of the Atonal superfamily control numerous aspects of differentiation for vertebrae organ development and function [25,26,27]. Atoh1 is a member of the bHLH family that plays a pivotal role in the differentiation of Paneth cells. Paneth cells are critical for providing stem cell niche to Lgr5+ cells in the intestinal crypts [28]. Atoh8 is the sole mammalian member of the bHLH factor that is a part of the NET family [29]. Atoh8 has been previously studied in the context of the differentiation of osteoblasts. In a recent study, Atoh8 was found to be induced by BMP signaling; BMP-induced Atoh8 regulated the RankL/OPG ratio indirectly via Runx2 to regulate osteoclast number and to maintain bone volume in mice [20]. In our preliminary experiment, RankL-treated intestinal organoids showed a significant increase in expression of BMP2 and BMP6 expression (Supplementary Figure S3A). However, Runx2 expression remained unchanged with RankL treatment. We also observed that Atoh8 expression could be induced by growing organoids in BMP2 and BMP6 alone without RankL treatment (Supplementary Figure S3B). However, as Runx2 was not activated by RankL, further investigation is required to find out if BMP2/BMP6 directly induces Atoh8 to control the density of the M cell population in the Peyer’s patch.

**Figure 6:**
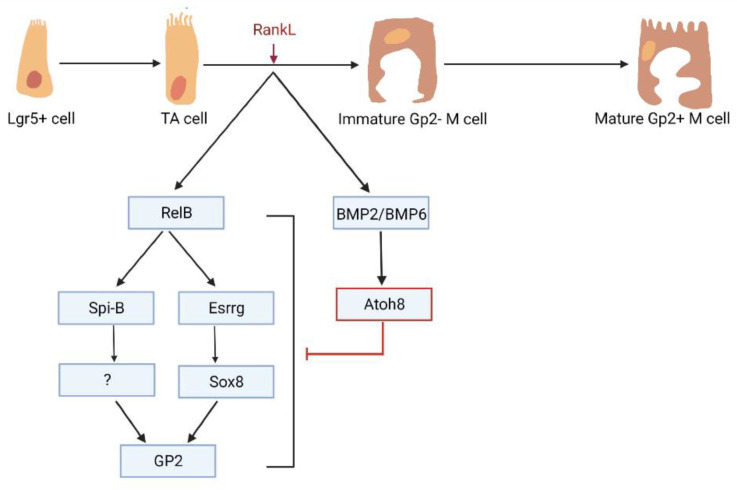
Atoh8 regulates the density of M cell population in the FAE. Loss of Atoh8 significantly increased the expression of M cell-associated transcription factors such as Spi-B, Sox8, Esrrg as well as early M cell expression markers. The population of Gp2+ M cells increased significantly in Atoh8 null mice.

Our experiments with Atoh8 intestinal-specific knockout mice exhibited a higher number of Gp2+ M cells when compared to the *VilCre* wildtype. Well-established early markers of M cells such as MarcksL1 and TNFAIP2 were all significantly upregulated. Spi-B, Esrrg, and Sox8, which are critical for the maturation of Gp2+ M cells and fall under the purview of RankL and Nfkb (both classical and non-canonical) signaling, were found to have higher transcriptional expression. This implies that BMP2/BMP6 induced Atoh8 regulates the signaling pathway that leads to Gp2+ M cell development and differentiation (Figure 6). Isolation of intestinal organoids and treatment with RankL indicated that Atoh8 deficiency that led to increased Gp2 was epithelium intrinsic. Previous research involving transcription factors involved in M cell differentiation like Spi-B and Sox8 showed that transcription factor deficient mice also exhibit reduced lymphoid cell population in PP along with reduced GP2 expression [16,17]. Osteoprotegerin (OPG) deficient mice showed higher Gp2+ M cells but also higher numbers for lymphoid cells in terms of population (Kimura et al., 2020). In the current study, the regulation of M cell density by Atoh8 did not affect mucosal lymphoid cell populations that have previously been shown to be mediated by IgA. More specifically, the population of lymphoid cells in the Peyer’s patch of Atoh8 *lox/VilCre* remained unchanged after weaning despite the increased Gp2+ M cell quantities. To account for delayed immune response/population changes, we additionally analyzed 10-week-old Atoh8 *lox/VilCre* mice and similarly observed unaltered populations of GC B and Tfh cells when compared to the control (Supplementary Figure S4). Nevertheless, in the older mice, a slight increase in the B and T helper (Th) cell frequencies could be seen. Importantly, we observed a higher transcytosis capacity of nanobeads into the Peyer’s patches of Atoh8 intestinal KO animals, twice the amount of uptake when compared to control mice. This indicates that Atoh8 *lox/VilCre* mice with higher Gp2 expression also had functional characteristics during an infection. However, further studies with *Salmonella typhimurium* infection or other pathogenic antigens would be required to better elucidate the differences in the immune response.

OPG binds to RankL and acts as a decoy receptor instead of Rank. OPG-deficient mice showed higher RankL secretion, more Gp2+ M cells, higher transcytosis capabilities, an increased population of systemic lymphoid tissues, and enhanced immune response [18]. As we looked to see if Atoh8 intestinal KO mice had increased RankL production, our flow cytometric analysis showed no increase in RankL+ T cells, indicating that Atoh8 acts independent of RankL signaling in the GALT. Although the OPG null mice showed higher M cells and enhanced immune responses, they were highly susceptible to infection by pathogenic bacteria. An increase in M cells could also increase the transcytosis of botulinum toxins and scrape prion protein into the body. [30] Therefore, considering that the lack of Atoh8 in the intestine led to an increase in M cells, it is reasonable to conclude that Atoh8 limits transcytosis of pathogenic agents by regulating the number of M cells. The delicate equilibrium of maintaining M cell density in the Peyer’s patch via Atoh8 may have been established over time as an evolutionary mechanism to control intestinal homeostasis in relationship to invasive antigens and mucosal immune responses. While Atoh1, a family member of Atoh8, regulates Paneth cell differentiation in the intestinal crypt via notch signaling, simultaneously inhibiting Paneth cell differentiation in the neighboring cell, it can be speculated that Atoh8 employs a similar mechanism in maintaining the population. However, further exploratory studies are needed to understand how the epithelial intrinsic Atoh8 regulates the population of M cells.

## 4. Materials and Methods

### 4.1. Mice

All animal experiments were approved by the Finnish National Animal Experiment Board (Permit: ESAVI/5824/2018). Mice were maintained on standard light–dark conditions, with food and water ad libitum at the pathogen-free animal facility of the Faculty of Medicine and Health Technology. B6.Cg-Tg(Vil1-cre)1000 Gum/J mice (Cat No: 021504) were purchased from Jackson Laboratories. Atoh8 lox/lox mice, in which exon 1 is flanked by two loxP sites, were provided by Rosa Gasa (Ejarque, et al. 2016). To generate intestinal deletion of Atoh8, Vil1-cre mice were bred with Atoh8 lox/lox. The F1 generation was backcrossed with Atoh8 lox/lox to generate mice homozygous for floxed Atoh8 allele carrying the Vil1-cre transgene. Littermates with Vil1-cre allele were used as control. Mice genotypes and Atoh8 deletion were confirmed by PCR with genotyping primers 5′ ATTGGAGGAAGGCTCGGTGAA 3′ and 5′ TTGGCATTCGTCGTGCTGTC 3′. Representative genotyping of PCR genotyping (Supplementary Figure S2).

### 4.2. Immunohistochemistry and Immunofluorescence

Peyer’s patches were isolated from the ileum and washed in cold PBS and embedded into paraffin blocks. Sections were cut from the blocks and rehydrated by washing with PBS. After blocking with 1% PBS/BSA supplemented with 5% normal donkey serum (Sigma -Aldrich, St. Louis, MO, D9663-10ML), antigen retrieval was performed with citrate buffer, pH 6.0 (121 °C for 5 min), and tissue sections stained overnight at 4 °C for Atoh8 (Thermo Fisher Scientific, PA5-20710, Waltham, MA, USA) antibody. Goat Anti-Rabbit was used for the secondary antibody (Thermo Fisher Scientific, A32731). Light microscopy was used for detection and analysis.

Intestinal crypt organoids were analyzed by whole-mount immunostaining, crypt-organoids were grown in an 8-well chamber plate and cultured for 4 days with and without RankL (100 ng/mL) after which they were fixed with 4% paraformaldehyde, followed by permeabilization with 0.1% Triton X-100. The organoids were stained with Gp2 (MBL, D278-3) antibodies overnight at 4 °C. This was followed by Anti-Rat for the secondary antibody. Gp2 expressing cells were analyzed by Nikon A1R+ Laser Scanning Confocal Microscope after mounting with ProLong Diamond with DAPI mounting solution (Molecular Probes P36962).

### 4.3. Isolation of Follicle-Associated Epithelial Cells (FAE) and Villous Epithelium Cells (VE)

Illeal PPs along with small pieces of intestine were isolated from the ileum of control mice and Atoh8 *lox/VilCre* mice. After flushing the tissues with cold PBS, they were incubated in 30 mM EDTA, 5 mM DTT in PBS, and gently shaken in ice on a rocker for 20 min. Surrounding epithelial cells were peeled off from lamina propria and PP’s. FAE was carefully cleaned off from surrounding VE tissues with a 26-gauge needle under a stereomicroscope. Trizol was added to the cleaned FAE and proceeded by RNA isolation.

### 4.4. Mouse Intestinal Organoid Culture

Mouse intestinal crypts were isolated and culture techniques were observed as previously described by Sato et al. (2011) and de Lau et al. (2012) [17,28]. Collected duodenum was washed in PBS and cut longitudinally, and villi were gently scraped off using glass slides. After further washes with PBS, the duodenum was cut into 2 mm pieces and pipetted up and down 4–6 times in 10 mL PBS using a 10 mL pipette. Once the suspension was relatively clear, the pieces were suspended in 10 mM EDTA in PBS for 20 min rocking at room temperature. A 70 μm cell strainer (Fisher Scientific, Waltham, MA) was used to strain the crypts from the rest of the epithelium. This mixture was enriched to crypt fraction through centrifugation at 150× *g* for 5 min. The crypts were cultured on a 24-well plate by embedding them in 30 ul of Matrigel (Corning, NY, USA). Organoids were cultured in an optimal medium consisting of advanced DMEM/F12 (Thermo Fisher Scientific) that contained HEPES (10 mM, Sigma-Aldrich, St. Louis, MO, USA), Glutamax (2 mM, Thermo Fisher Scientific), Penicillin–streptomycin (100 U/mL, Sigma-Aldrich-Aldrich, St. Louis, MO, USA), B-27 supplement minus Vitamin A (Thermo Fisher Scientific), *N*-2 supplement (Thermo Fisher Scientific), *N*-acetylcysteine (1 mM; Sigma-Aldrich, St. Louis, MO, USA), recombinant WNT (100 ng/mL R&D Biosystems, Minneapolis, MN, USA), recombinant murine EGF (50 ng/mL; Thermo Fisher Scientific), recombinant murine Noggin (50 ng/mL; PeproTech Cranbury, NJ, USA), Chir99021 (3µm, Selleckchem, Houston, TX, USA) recombinant mouse R-spondin1 (1 μg/mL; R&D BioSystems). Media were changed every 2 days. For M cell differentiation, recombinant mouse RankL (100 ng/mL, Peprotech) was added to the media and incubated for 4 days. To check for Atoh8 expression via BMP2/BMP6, organoids were grown in EGF, R-spondin, and BMP2 (100 ng/mL, R&D Biosystems) and EGF, R-spondin, and BMP6 (100 ng/mL, R&D Biosystems) according to the protocol of Calpe et al [31]. Noggin was not added for the BMP2/BMP6 experiments as it acts as an antagonist against BMP signaling.

### 4.5. CRISPR–Cas9 Gene Knockout of Intestinal Organoids

Guide RNAs for the Rank gene were designed using CRISPR design tool (http://crispr.mit.edu, accessed on 7 April 2019) (Shalem, O. et al. Science 343, 84–87 (2014)). The guides were cloned into the lentiCRISPR v2 vector (Addgene, Watertown, MA, USA) and the cloned product was transfected into HEK 293FT cells (ThermoFisher, R7007). After 48 h, the supernatant was collected and concentrated with Lenti-X concentrator (Clontech Mountain View, CA, USA). The 293FT cell line was tested for mycoplasma. Intestinal organoids were grown in ENCY (EGF, Noggin, Chir-99021, and Y-27632) 2 days before transduction. After dissociating organoids into single cells using TrypLE Express (Thermo Fisher Scientific) supplemented with 1000 U/mL DnaseI for 5 min at 32 °C, the cells were washed once with Advanced DMEM and resuspended in transduction medium (ENR media) supplemented with 1 mM nicotinamide, Y-27632, Chir99021, 8 μg/mL polybrene (Sigma-Aldrich) and mixed with the concentrated virus. The mixture was centrifuged for 1 h at 600× *g* 32 °C followed by 3 h incubation at 37 °C, after which they were collected and plated on 60% Matrigel overlaid with enriched transduction medium without polybrene. On day 2 and day 4, transduced organoids were selected with 2 μg/mL of puromycin (Sigma-Aldrich), after which clones were expanded in maintenance ENR medium. Knockout was confirmed by Western blot.

Oligonucleotides used for generation of gRNAs for Rank CACCGAAAGCTAGAAGCACACCAG, AAACCTGGTGTGCTTCTAGCTTTC.

### 4.6. Real-Time Quantitative Reverse Transcription PCR

Total RNA was isolated from intestinal organoids, FAE, and VE tissues from mice using TRIzol (Life Technologies Carlsbad, CA, USA). iScript cDNA synthesis Kit (Biorad, Hercules, CA, USA) was used to transcribe the isolated RNA to the first-strand cDNA. qPCR amplification was detected using SsoFast EvaGreen Supermix (Biorad, Hercules, CA172-5203) and a CFX96 detection system (Biorad). The specific primers used, are listed in the supplementary data (Table S1).

### 4.7. Whole-Mount Immunostaining of M Cells in FAE

Ileal PPs were dissected from the small intestine and transferred to a 10 cm dish containing 30 mL cold PBS. Excess intestinal tissues around the FAE were cut and removed under a stereomicroscope using forceps. PP’s were washed sufficiently by using a 1 mL syringe with a 26-gauge needle under a stereomicroscope. The mucous layer on the FAE should be flushed out with a water stream to prevent background noise detection. PPs were transferred to the 1.5 mL tube containing 1 mL PBS, washed by vortexing and the supernatant was discarded. After 3 washes, 300–1000 µL Cytofix/Cytoperm buffer (BD Biosciences, Franklin Lakes, NJ, USA) was used for blocking and permeabilization for 25 min at room temperature. Perm/wash buffer (BD Biosciences, Franklin Lakes, NJ, USA) was used to wash the PPs after which they were stained with PE-conjugated anti-GP2 antibody (MBL; 1:10 in Perm/Wash buffer) overnight at 4 °C. Following the primary antibody staining, PPs were washed 3 times with wash/perm buffer and stained for 30 min at RT in Alexa Fluor 546-conjugated anti-Rat IgG (Thermo Fisher Scientific, Waltham, MA, USA). The PPs were washed again 3 times with wash/perm buffer and mounted with ProLong Diamond with Dapi mounting solution (Molecular Probes P36962). Slides were examined with a laser scanning confocal microscope (Zeiss LSM 800 LSCM).

### 4.8. Flow Cytometry

Peyer’s patches from *VilCre* mice and Atoh8 *lox/VilCre* animals were isolated from the ileal section. The tissues were washed in PBS and incubated in 5 mL Spleen Dissociation Medium for 15–20 min at 37 °C while vigorously shaking at 250 rpm. To generate a single cell suspension, PPs were placed on a sterile (autoclaved) 70 μm nylon mesh cell strainer and ground into the mesh using the base of a plunger from a 1 cc syringe. The suspension was incubated in 1 mm EDTA for 5 min in a rocker after which the suspension was passed through a 70 μm nylon mesh cell strainer. After the isolation step, the single-cell suspensions were prepared with FVS510 viability stain (#564406; Becton, Dickinson and Company, Franklin Lakes, NJ, USA) and CD16/CD32 Monoclonal Antibody (#16-0161-85; Thermo Fisher Scientific,). Surface staining was done using fluorochrome-conjugated anti-mouse antibodies against CD3e, CD8a, CD4, B220, PD1, CXCR5, GL7, FAS, IgA, and RANKL (Thermo Fisher Scientific). To prevent spectral overlap of emitted fluorescence, cells were divided into replicates, and two separate antibody panels were used in the staining procedure. Flow cytometry was done with FACSAria™ Fusion (Becton, Dickinson and Company), and data were analyzed with FlowJo (v. 10.6.1, Tree Star, OR, USA). Statistical analyses were done with Prism v. 5.02 (GraphPad Software, San Diego, CA, USA) calculated using a two-tailed *t*-test. *p* values of < 0.05 were considered significant.

### 4.9. Quantification of Transcytosis of Fluorescent Beads by M cells

Atoh8 *lox/VilCre* and *VilCre* mice fasted for 3 h and 1011 of 200 nm diameter polystyrene nanoparticles (09834-10, Fluoresbrite YG; Polysciences Warrington, PA, USA) were orally administered via oral gavage. After 4 h, two PPs were collected from the ileum and jejunum and fixed with 3.7% formalin/PBS for 2 h. Fixed tissues were incubated overnight with 30% sucrose in PBS and finally embedded in the OCT compound (Sakura Fintech, Torrance, CA, USA). Ten sequential 15 µm sections were cut and examined by fluorescence microscopy. The number of fluorescent particles transcytosed was counted manually.

### 4.10. Statistical Analyses

Results were benchmarked and analyzed by using an unpaired two-tailed Student’s *t*-test. Statistical significance was set at *p* < 0.05 throughout all experiments. All statistical analyses were measured by using GraphPad Prism 6 software. No statistical methods were used to determine the sample size. These experiments were not randomized, and the investigators were not blinded to allocation during experiments and outcome assessment.

## Data Availability

Not applicable.

## Supplementary Materials

The following are available online at https://www.mdpi.com/article/10.3390/ijms22179355/s1.

## Abbreviations

M cells	Microfold cells
GALT	Gut-associated lymphoid tissues
PP	Peyer’s Patch
FAE	Follicle associated epithelium
VE	villous epithelium
RankL	Receptor activator of nuclear factor kappa B ligand
Rank	Receptor activator of nuclear factor kappa B
PRC2	polycomb repressive complex 2
Esrrg	Estrogen-related receptor gamma
