# Supplementary material for: PRC2 Regulated Atoh8 Is a Regulator of Intestinal Microfold Cell (M Cell) Differentiation
Source: Int J Mol Sci. 2021 Aug 28;22(17):9355. doi: 10.3390/ijms22179355 (PMC8431250; doi:10.3390/ijms22179355)
Supplement: Supplementary file 1 [file ijms-22-09355-s001.zip › ijms-1292728-supplementary.pdf]

International Journal of *Molecular Sciences*

MDPI

Supplementary file

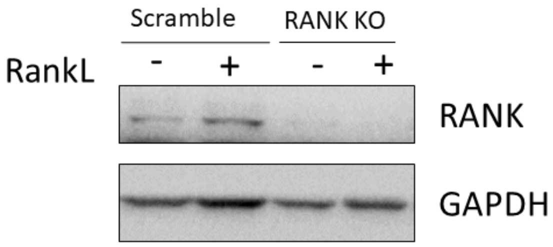

Figure S1. Immunoblot analysis of CRISPR generated RANK KO. RANK KO validation by immunoblot analysis of RANK KO and Scrambled intestinal organoids generated by CRISPR-Cas9 genome editing in C57BL/6rj intestinal organoids. These organoids were grown with and without RankL 100ng for 4 days. Data from Rank KO organoids also published in 10.1016/j.jcmgh.2021.05.014 .

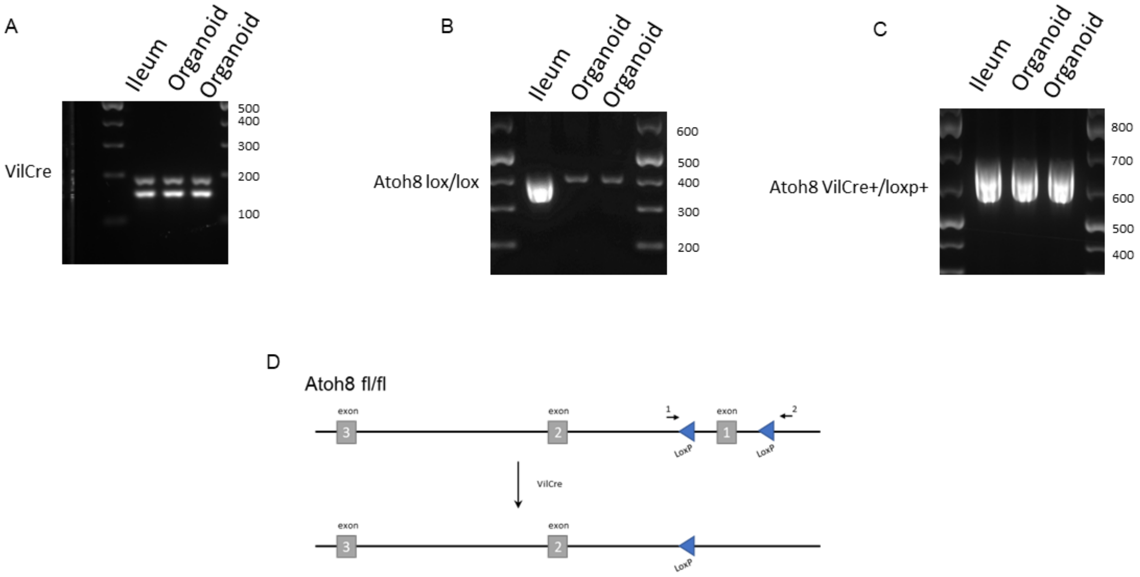

Figure S2. Representative genotyping PCR amplification of intestinal *VilCre*, Atoh8 *flox/flox* wild-type and Atoh8 *VilCre/loxp*. **A**) PCR for detection of VilCre recombinase is shown. Upper band: internal control (182 bp). Lower band: VilCre transgene (150 bp). 100 bp molecular marker brightest line corresponds to 500 bp. **B**) Floxed allele 400 bp. **C**) Genotyping of Atoh8 *lox/VilCre* 600bp. **D**) Scheme and representative PCR amplification verifying Cre-mediated recombination of the Atoh8 floxed allele in the intestine.

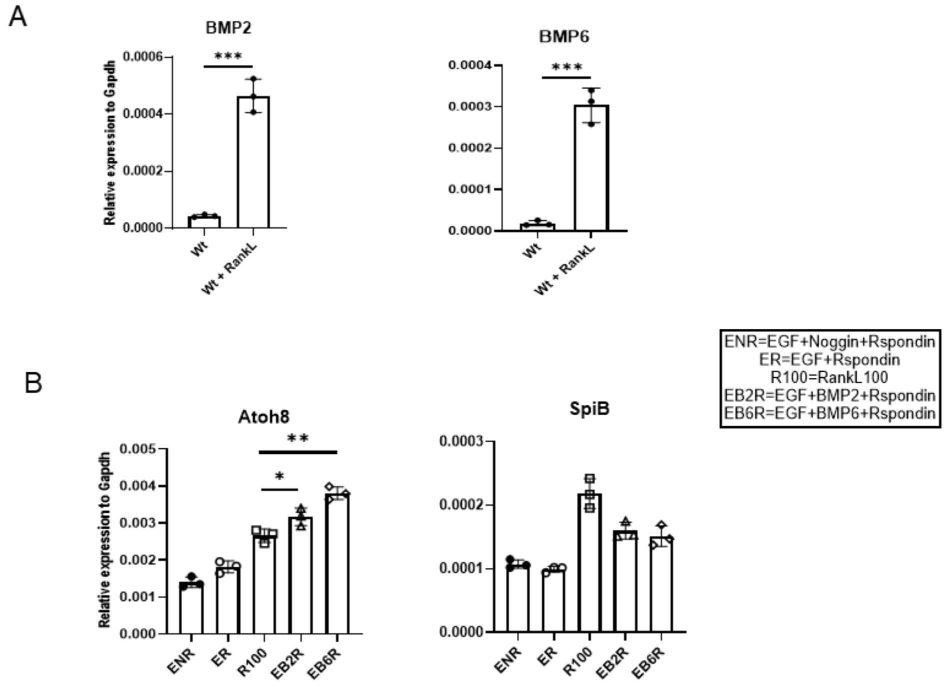

Figure S3. Atoh8 signaling is mediated by RankL/BMP2-BMP6 signaling**.** (**A**) Organoids from small intestinal crypts of wildtype mice were cultured with or without RANKL for 3 d. (**A**) qPCR analysis of M cell–associated genes expressed in the organoid cultures. Values are presented as the mean ± SD; *** $p < 0.005$; unpaired two-tailed Student's *t* test, $n = 3$. Data are representative of two independent experiments. (**B**) Organoids isolated Wt mice were grown for 3 days in EGF, Noggin, Rspondin media, EGF, Rspondin media, 100ug of Rankl media, Egf, BMP2 Rspondin media and Egf, BMP6, Rspondin media. Values are presented as the mean ± SD; *, $p < 0.05$, **, $p < 0.001$; unpaired two-tailed Student's *t* test, $n = 3$. Data are representative of two independent experiments.

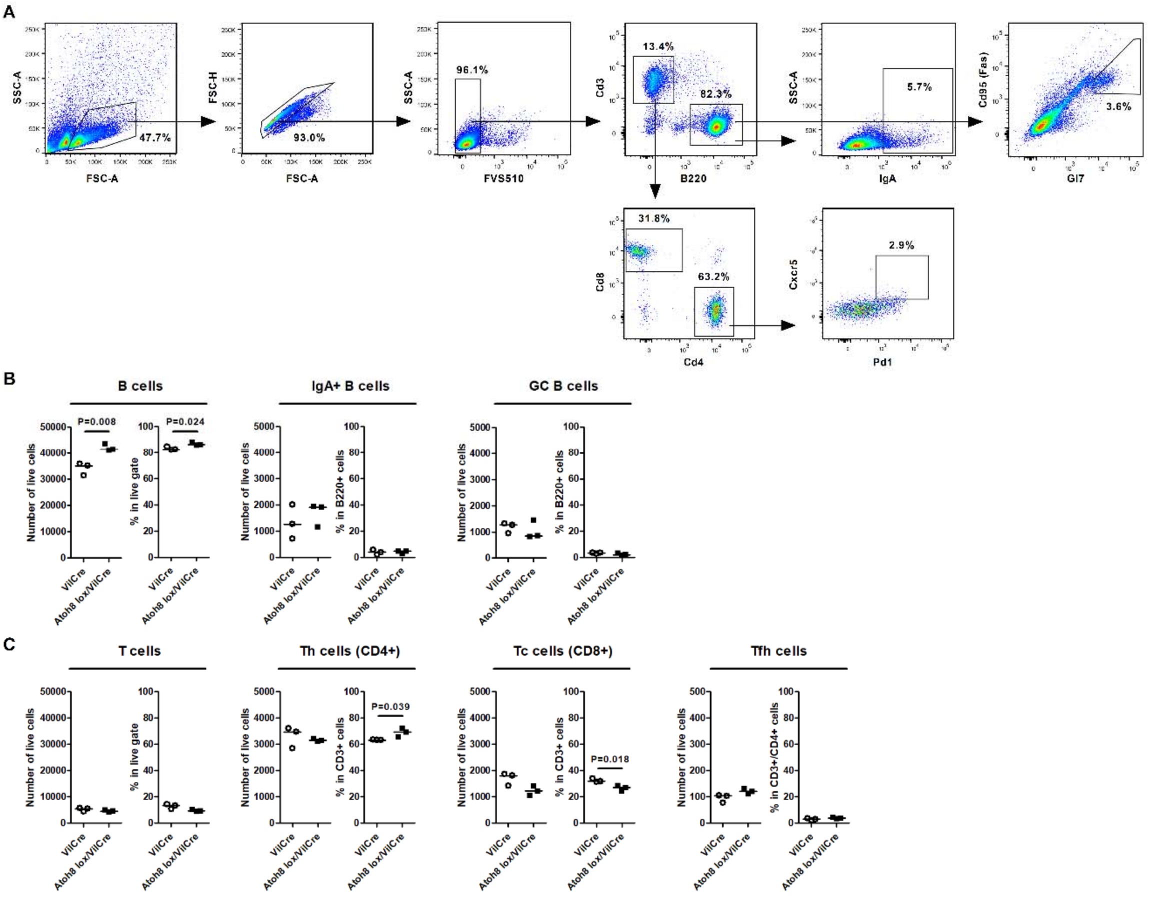

Figure S4. The loss of Atoh8 did not change the composition of lymphoid cells in 10-week-old mice. (**A**) Gating scheme for analysis of B and T cell populations in *VilCre* and Atoh8 *lox/VilCre* ileal PPs. **B** (CD3ε−B220+) cells were analyzed for IgA+ B and CD95+GL7+ GC B cells. T (CD3ε+B220−) cells were analyzed for total CD4+ Th, CD8*α*+ cytotoxic T (Tc), and CD4+CD8*α*−CXCR5+PD-1+ Tfh cells. (**B** and **C**) Flow cytometry analysis of indicated immune cells in ileal PPs. (**C**) Number of total B cells, GC B cells, and IgA+ B cells. (**D**) Number of total T cells, Tc cells, Th cells, and Tfh cells; n.s., not significant; Student's *t* test, *n* = 3 per group.

Table S1. List of primers.

Oligonucleotide	Sequence (5' to 3')
Gapdh_fwd<br>Gapdh_rev	TGTGTCCGTCGTGGATCTGA<br>CCTGCTTCACCACCTTCTTGA
Spi-B_fwd<br>Spi-B_rev	GGAGTCTTCTACGACCTGGACAG<br>GCAGGATCGAAGGCTTCATAGG
Sox8_fwd<br>Sox8_rev	GGACCAGTACCCGCATCTCC<br>TTCTTGTGCTGCACACGGAGC
GP2_fwd<br>GP2_rev	GTGTACAAGTTACAGGGTACCCC<br>GACAAGTAATCTCACAATTCTTGG

CCL9_fwd<br>CCL9_rev	GCCCAGATCACACATGCAAC<br>AGGACAGGCAGCAATCTGAA
MarcksL1_fwd<br>MarcksL1_rev	CCCGTGAACGGAACAGATGA<br>CCCACCCTCCTTCCGATTTC
Esrrg_fwd<br>Esrrg_rev	GTGTCTCAAAGTGGGCATGC<br>GCTGTTCTCAGCATCTATTCTGC
Aif1_fwd<br>Aif1_rev	GGATTTGCAGGGAGGAAAA<br>TGGGATCATCGAGGAATTG
CCL20_fwd<br>CCL20_rev	TGTACGAGAGGCAACAGTCG<br>TCTGCTCTTCCTTGCTTTGG
TNFAIP2_fwd<br>TNFAIP2_rev	GTGCAGAACCTCTACCCCAATG<br>TGGAGAATGTCGATGGCCA
18s rRNA_fwd<br>18s rRNA_rev	GTAACCCGTTGAACCCCATT<br>CCATCCAATCGGTAGTAGCG
Atoh8_fwd<br>Atoh8_rev	CGGGGGAAAGTTCCTACTCGTC<br>CGGAAGAATCCGGGTGGTTATT
OPG_fwd<br>OPG_rev	ACCCAGAAACTGGTCATCAGC<br>CTGCAATACACACACTCATCACT
